# Kabat Rehabilitation in Facial Nerve Palsy after Parotid Gland Tumor Surgery: A Case-Control Study

**DOI:** 10.3390/diagnostics12030565

**Published:** 2022-02-23

**Authors:** Ciro Emiliano Boschetti, Giorgio Lo Giudice, Chiara Spuntarelli, Carmine Apice, Raffaele Rauso, Mario Santagata, Gianpaolo Tartaro, Giuseppe Colella

**Affiliations:** 1 Oral and Maxillofacial Surgery Unit, Multidisciplinary Department of Medical-Surgical and Dental Specialties, University of Campania “Luigi Vanvitelli”, Via Luigi de Crecchio, 6, 80138 Naples, Italy; ciroemilianoboschetti@gmail.com (C.E.B.); chiaraspunta@hotmail.it (C.S.); raffaele.rauso@unicampania.it (R.R.); mario.santagata@unicampania.it (M.S.); gianpaolo.tartaro@unicampania.it (G.T.); giuseppe.colella@unicampania.it (G.C.); 2 Maxillofacial Surgery Unit, Department of Neurosciences, Reproductive and Odontostomatological Sciences, University of Naples “Federico II”, Via Pansini, 5, 80131 Naples, Italy; 3 EY—AI & RPA Center of Excellence, Via Meravigli 12/14, 20123 Milan, Italy; carmine.apice@it.et.com

**Keywords:** Kabat, facial nerve palsy, FNP, facial nerve paralysis, salivary gland surgery

## Abstract

Temporary facial nerve palsy after parotid tumor surgery ranges from 14 to 65%, depending on surgery, tumor type, and subsite. The study aimed to evaluate the role of Kabat physical rehabilitation in the outcomes of patients affected by severe facial nerve palsy following parotid gland surgery. The results and clinical data of two groups, Kabat and non-Kabat (control), were statistically compared. Descriptive statistics, the multiple linear regression model, difference in difference approach, and the generalized linear model were used. F-Test, Chi-square test, McFadden R-squared, and adjusted R-squared were used to assess the significance. The results showed that the House–Brackmann (HB) stage of patients who had physiotherapy performed were lower than the control group. The decrease of HB staging in the Kabat group at 3 months was −0.71 on average, thus the probability of having a high HB stage decreased by about 13% using Kabat therapy. The results are statistically significant, and indicated that when the Kabat rehabilitation protocol is performed, mainly in the cases of a high-grade HB score, the patients showed a better and faster improvement in postoperative facial nerve palsy.

## 1. Introduction

Facial nerve palsy (FNP) is a disabling pathology that significantly affects the functional, psychological, social, and occupational aspects of a patient’s life. This condition represents a frequent complication after parotid gland surgery, regardless of the surgical technique used and despite the most recent advances in surgical instrumentation and intraoperative monitoring [1,2]. The literature is unclear on the specific incidence of temporary FNP after parotid tumor surgery, and has been shown to range from 14 to 65% depending on the surgery, tumor type, and subsite [3,4].

In order to assess FNP severity, multiple scales have been developed such as the House–Brackmann (HB), Sunnybrook, or Yanagihara facial nerve grading scales [5,6]. The HB scale, which is the most adopted in the United States and Europe, is divided into six grades, from normal function to total palsy.

While the literature agrees that any treatment should be implemented as it overall improves the patients’ quality of life, each to a different extent, no specific medical, surgical, or physiotherapy guidelines are currently available for post-operative FNP [7].

Updated guidelines have confirmed steroids to be effective at increasing the possibility of complete facial functional recovery in acute FNP, while novel treatments such as nimodipine and mycophenolate mofetile are under study. Nonetheless, postoperative corticosteroid treatment for iatrogenic FNP is unadvised, although clear evidence is lacking [8,9,10,11].

The literature describes the benefits of physiotherapy in nerve recovery (led either by a professional or home-based), and further studies have analyzed the aid given by facial taping and low-level laser therapy [12,13,14,15].

Kabat rehabilitation therapy is a treatment based on proprioceptive neuromuscular facilitation (PNF), a concept currently applied in orthopedic pathologies, post-stroke management, systemic sclerosis, and FNP rehabilitation [16,17,18,19,20]. This physiotherapy protocol is based on proprioceptors stimulation, by applying pressure in combination with traction movements, it is able to evoke and restore the neuromuscular circuits, recovering the normal function of the nerve endings in the muscles [21].

The aim of this study was to retrospectively evaluate the role of Kabat physical rehabilitation in the outcomes of patients affected by severe FNP following parotid gland surgery. Clinical data were compared between two different groups: a non-Kabat group and Kabat group.

## 2. Materials and Methods

This study was conducted retrospectively by evaluating the patients’ medical reports treated at the Maxillo-Facial Surgery Unit of the University of Campania “Luigi Vanvitelli”. The procedures were in accordance with the Helsinki Declaration, and the study was approved by the internal Ethical Committee (Prot. N. 313, 23 October 2020).

Patient records were selected according to the following inclusion criteria: patients with parotid gland tumor diagnosis, diagnosis of FNP after surgery, and HB stages IV-V recorded at 7 days follow-up.

The exclusion criteria were as follows: patients with preoperative facial palsy, patients that underwent postoperative radiotherapy, patients who started Kabat therapy more than seven days after surgery, patients with severe comorbidities, and/or those affected by pathologies that could interfere with the evaluation (neurodegenerative diseases or autoimmune diseases).

We collected clinical and pathological data (gender, age, pathology, tumor subsite, extent of surgery, and HB score) from 425 patients who underwent parotid gland surgery between January 2010 and March 2019 (Table 1).

Out of 425 patients, 56 met our criteria: HB IV (34 subjects) and HB V (22 subjects). All subjects performed preoperative clinical examination, salivary glands ultrasonography, magnetic resonance imaging (MRI) with contrast enhancement, and fine-needle aspiration cytology with ultrasonography guide if indicated. The extent of surgery, extracapsular dissection (ED), partial parotidectomy (PP), superficial parotidectomy (SP), or total parotidectomy (TP) depended on the location, tumor size, and histological type. One head surgeon conducted the surgical procedures. Intraoperative facial nerve monitoring and surgical magnification using surgical loupes were used to identify the nerve. A single physician routinely evaluated the functional status of the facial nerve using the HB grading system at 7 days, and 1, 3, and 6 months post-operative. The evaluation was determined by measuring the excursion movements of the eyebrow and the angle of the mouth: 1 point was given for each 0.25 cm movement, to a maximum of 1 cm, and a final score ranging from 0 (total paralysis) to 8 points (normal function), which was then classified accordingly [5]. A physical rehabilitative protocol was employed according to the method proposed by Kabat, starting on day 7, at the time of suture removal, continuing three times per week for 12–24 weeks.

During the Kabat rehabilitation session, the patients performed specific diagonal and spiral movements, involving the following three muscle fulcrums:

- Upper fulcrum: includes the corrugators, frontalis, and orbicularis;
- Intermediate fulcrum: includes the common elevator muscle of the upper lip and wing of nose, the dilator naris, and the mitriform;
- Lower fulcrum: includes the risorius, zygomaticus major, the orbicularis, the zygomaticus minor, the triangular of the lower lip, buccinator, chin muscle, and square muscle of the chin [22].

The patients were split into two groups for the analysis: Kabat and non-Kabat (control). The former consisting of 28 of the 56 patients who had followed our therapeutic protocol and the latter consisting of patients who did not follow it, proceeding with standard therapies for the management of the rehabilitation cycle (28 patients). The two groups were homogeneous in terms of gender, age, HB grade, and extent of surgery, and we statistically compared the results and clinical data obtained (Table 2).

### Statistical Analysis

The statistical analysis and figures were produced using R (R Core Team, 2014). Descriptive statistic was used to evaluate the differences between the two groups and the relation between age and HB grading. A multiple linear regression model was adopted to evaluate a possible relationship between HB grade at 3 months and other variables present in our dataset. The difference in difference approach, typical of the Quasi-experimental design, was used to determine the effects of therapy on HB grading in both groups. F-Test and R-squared were used to assess the significance of our model. A logistic regression model for binary data was used, dividing the variables of interest into the following two groups:

- People with a mild grading (from I to II) of HB registered at 3 months;
- People with a severe grading (from III to V) of HB registered at 3 months.

This model was further used considering two variables: age and Kabat therapy. Chi-square test and McFadden R-squared were used to assess the significance of our model.

## 3. Results

### 3.1. Descriptive Statistics

**Figure 1:**
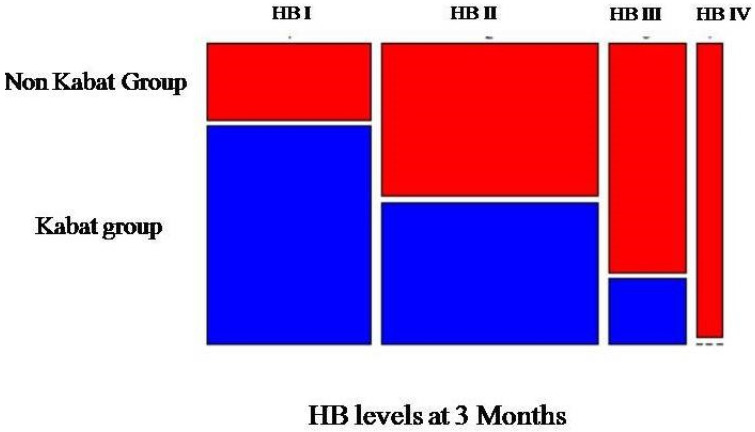
Graphical representation of the collected data.

Kabat and non-Kabat (control) groups were homogeneous in terms of gender (20 males and 8 females), mean age (53.8 ± 18.3 vs. 54 ± 21.6), HB grade at 7 days, and extent of surgery. Our analysis was focused on the effects of Kabat considering the median position (3 months), as the other follow-up records could be perceived to be too close or too far from the surgical intervention. The graph shows that there are different compositions in HB grading, depending on whether the patients have followed Kabat therapy or not (Figure 1).

**Figure 2:**
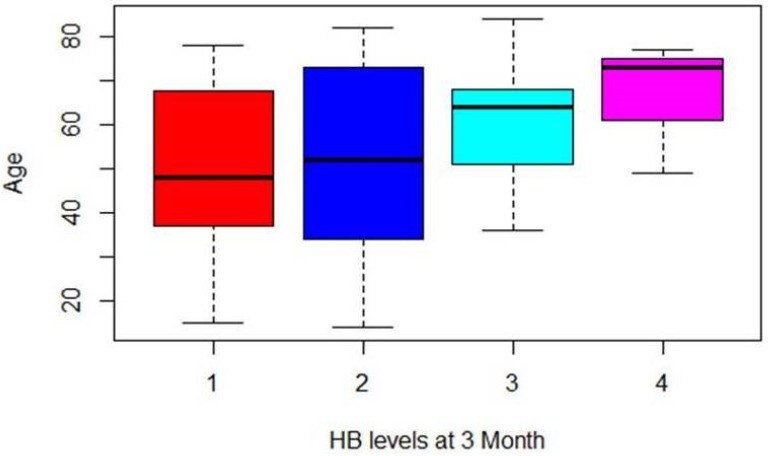
Graphical representation of the relation between age and HB score of the entire cohort of patients.

The percentages of patients who had not used the therapy increased compared to the HB grading recorded. Regarding the relation between age and HB grading, it is possible to assume that postoperative recovery could be longer for older subjects than younger ones. A positive relation was shown: older people had a higher HB grading on average (Figure 2).

### 3.2. Multiple Linear Regression Model

The results of the multiple linear regression model showed the relation between the results and the Kabat therapy to be statistically significant (*p* < 0.001) (Table 3).

We grouped the extent of surgery variable, collapsing this in two levels, in order to divide people that received a total parotidectomy and those who did not. In this case, the extent of surgery was still not useful to explain our response variable. As the role played by the other variables was not significant, we decided to remove gender and extent of surgery from the dependent variables (Table 4).

The decrease in HB grading in the Kabat group at 3 months was −0.71 on average. The difference in difference approach result was almost identical to the one obtained with the linear regression model (−0.714286) (*p* = 0.0006502; adjusted R-squared = 0.2133).

#### Generalized Linear Model

The results of the logistic regression model are shown in Table 5.

The odds ratio evaluation showed that the probability of having a high HB grading decreased by about 13% using Kabat therapy. Moreover, for each additional year of age, the odds of having a severe HB grading increased by about 3% ceteris paribus. The results were statistically significant, as shown by R-squared of McFadden (0.18) and Chi-Square tests (*p* = 0.0057).

**Figure 3:**
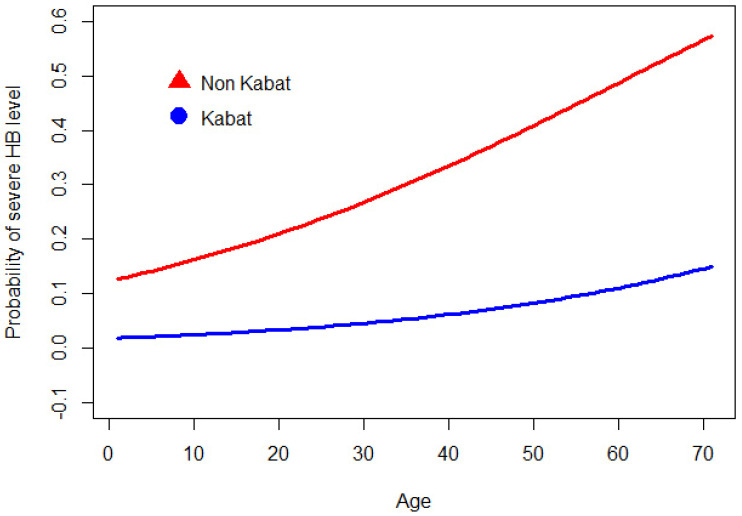
Profiles of patients at different ages using and not using Kabat therapy.

Using the parameters obtained from the model and to provide a graphical intuition of the results, we plotted the profiles of the patients at different ages, using and not using Kabat therapy (Figure 3).

**Figure 4:**
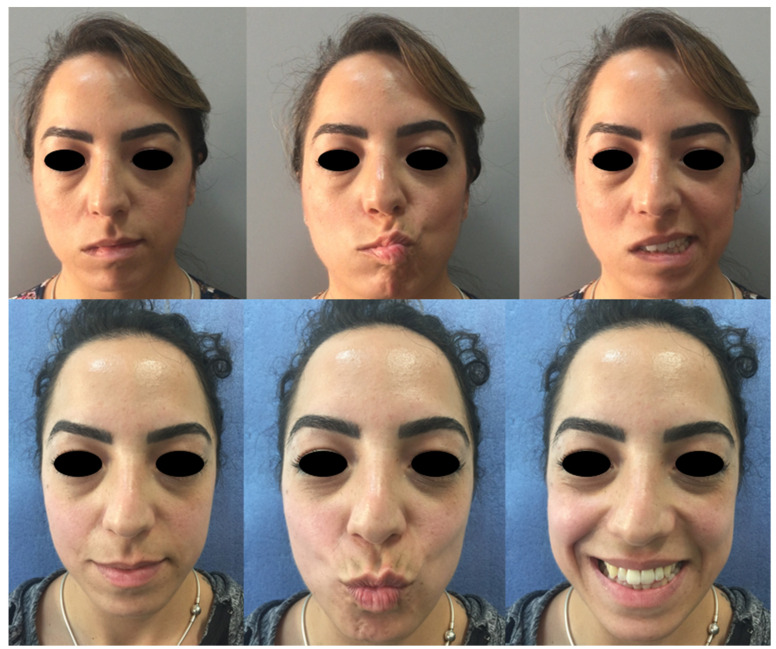
Patient 1. Clinical assessment of postoperative facial nerve palsy 7 days after surgery (**upper row**). Three month follow-up after Kabat therapy (**lower row**).

**Figure 5:**
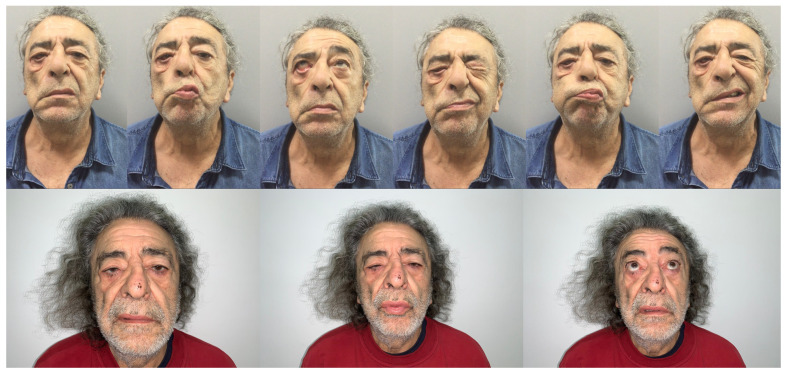
Patient 2. Postoperative facial nerve palsy 7 days after surgery (**upper row**) with noticeable right eye lagophthalmos. At the 3 month follow-up after Kabat therapy, right eye lagophthalmos was resolved (**lower row**).

For example, at age 60, the probability of having severe facial nerve palsy (HB III, IV, or V at 3 months) is 0.08 if the patient follows Kabat therapy, while the same probability increases up to 0.39 if the patient does not (Figure 4 and Figure 5).

## 4. Discussion

For head and neck surgeons, preservation of facial nerve integrity and function is a primary goal of parotid gland surgery, and still represents a major challenge. An occurrence of facial nerve palsy, despite the macroscopic continuity of the nerve, is not always tolerated, because of the possible impact on the quality of life, and the involvement of functional and aesthetic aspects (labial incontinence with drooling, chewing impairment, ectropion, keratoconjunctivitis, and severe facial asymmetry).

While authors have described the efficacy of Kabat rehabilitation in FNP from different etiologies, post-surgical studies are lacking. This study aimed to retrospectively evaluate the effects of the Kabat rehabilitation protocol that we routinely prescribed in patients with severe facial nerve deficiency (HB IV-V) after parotid gland surgery. A fair number of patients refused our physiotherapy prescription due to lacking financial resources to perform private therapy or no time available, thus allowing us to create a control group and evaluate the overall efficacy.

The facial nerve function was assessed and recorded 7 days, and 1, 3, and 6 months post-operative. The clinical outcomes recorded after 3 months could be considered the most interesting as they represent the median position compared to the other records (which could be considered either too close or too far from the surgery). Therefore, our analysis was focused mainly on the effects of Kabat at this time.

The graph in Figure 1 shows that there were different compositions in the grading of HB depending on whether the patients followed Kabat therapy or not. We observed that the percentages of patients who did not use the therapy increased compared to the HB grading recorded. This evidence supports the hypothesis that Kabat rehabilitation may have had, in statistical terms, a negative effect on the severity of facial nerve palsy.

The results shown in the multiple linear regression model and the difference in difference approach support the fact that rehabilitation is effective at decreasing high grading of HB (Table 3 and Table 4). The results of the generalized linear model show that HB grading decreased when using Kabat therapy (Table 5).

In terms of our understanding of the effectiveness of the therapy, it is also valuable to mention that both the variables of age and Kabat had no significance and there was a less significant effect on the measurement of HB grading at six months. This drove us to the conclusion that, despite the fact that the recovery of the patients would occur in any case after a certain time, Kabat therapy can accelerate the recovery process.

After physical rehabilitation, the degree of improvement (grade reduction at the HB) was significant in comparison to the final condition of the control group. Most of the patients treated with rehabilitative therapy recovered fully (HB I), while for a significant part of the control group patients, the maximum recovery was HB II. Furthermore, considering the follow-up period, a substantial improvement in HB grading was already appreciated after 3 months in patients undergoing Kabat rehabilitation. In fact, 45% of HB V subjects had an outcome at HB I compared to the non-Kabat group, in which no one showed the same improvement.

When considering the role played by gender, the extent of surgery, and the improvement of HB grade, no relation was found in both groups (Table 3).

On the other hand, assessing the age of the patients, the rate and the time of recovery were influenced negatively. Several studies have shown similar results, finding that age is a reliable prognostic factor for the final outcome [23]. In both groups, the older patients showed a higher final HB grade and also a longer time of recovery, probably due to the obvious differences in face tone between young and old subjects [13]. The older rehabilitated patients recovered to a lower final grade, with a shorter time of recovery with respect to the non-Kabat patients of the same age.

The overall results show how the use of Kabat significantly reduced the severity of facial nerve palsy measured on the HB scale (Figure 3).

Kabat physical rehabilitation induced an increase in facial muscle tone in the affected side and on the contralateral one, with functional and esthetic improvements. The rehabilitation, as previously reported in the literature, when applied at an early stage, produced better and faster recovery [23].

In our study, excellent outcomes in rehabilitated patients already 3 months after surgery were observed, and early rehabilitative therapy was always recommended.

The patient must be psychologically supported and encouraged to follow post-operative therapeutic indications for rapid functional recovery [23,24,25]. Compliance to Kabat therapy is pivotal for its success, as it is for every physiotherapy regimen [26,27]. Moreover, as facial palsy may be impactful on the patients’ social life, patients that notice substantial improvements between each follow-up are more prone to adhere to the physiotherapy regimen. Physicians may show comparison pictures and videos between follow-ups to let the patient acknowledge the improvements made, thus boosting the autonomous motivation and adherence to the therapeutic protocol. As a fair number of patients refused Kabat rehabilitation, we suggest providing an easily readable pamphlet, accompanied with explicative images, on how to perform Kabat therapy in a home-based fashion. We believe that such a compromise could be of help to patients and would promote satisfactory results, despite professional physiotherapy providing better outcomes.

Despite the promising results, this research had some limitations. The retrospective design of this study was chosen due to the availability of older HB scoring data in our database; lack of photo or video documentation for older cases did not allow us to perform a comparison using alternative, less subjective, scoring scales. The analysis provided by the House–Brackman scoring could be substituted using scoring systems such as Sunnybrook or eFace scale in further studies [6,28]. Facial nerve function analysis should provide both a static and dynamic evaluation of the condition. In fact, the Facial Nerve Grading Scale 2.0 was created as a revision of the HB scale, as a regional scale where the examiner assesses the function and synkinesis of four regions, which is then converted to a House–Brackmann scale grade, with consistent interobserver variability being reported [29]. Such low interobserver and intraobserver variability was also reported for the Sunnybrook Facial Grading Scale, retaining a high sensitivity and being able to track changes over time [30].

## 5. Conclusions

The results showed that early Kabat rehabilitation allowed for faster recovery of postoperative FNP diagnosed at any HB score. The improvement shown by patients affected by severe FNP (IV-V HB grade) suggests that early physiotherapy prescription might effectively reduce functional, aesthetic, and psychological sequelae.

## Figures and Tables

**Table 1:** Patients demographics.

No. (%)		Total Patients (*n* = 425)	HB IV (*n* = 34)	HB V (*n* = 22)
Age (Years, Mean, Range)		46.7 (12–86)	47.4 (14–84)	63.9 (36–82)
**Gender**	Male	254	24	16
	Female	171	10	6
**Pathology**	Benign tumors	352	29	20
	Malignant tumors	73	5	2
**Tumor subsite**	Superficial to the facial nerve	293	21	7
	Deep to the facial nerve	47	6	6
	Superficial and deep location	85	7	9
**Extent of surgery**	Extracapsular dissection	184	7	1
	Partial parotidectomy	122	9	3
	Superficial parotidectomy	73	15	7
	Total parotidectomy	46	3	11

**Table 2:** Subject characteristics in the Kabat and non-Kabat (control) groups and House–Brackman score at 7 days (7 D), 1 month (1 M), 3 months (3 M), and 6 months (6 M). ^a^: extracapsular dissection; ^b^: partial parotidectomy; ^c^: superficial parotidectomy; ^d^: total parotidectomy).

Patient	Gender	Age	Exent of Surgery (E.D ^a^, S.P ^b^, P.P ^c^, P.T ^d^)	HB (7 D)	HB (1 M)	HB (3 M)	Hb (6 M)
**Kabat Group**							
1	M	49	T.P	V	III	I	I
2	M	33	S.P	IV	III	II	I
3	M	52	P.P	V	IV	II	II
4	M	84	P.P	IV	IV	III	I
5	M	69	E.D	V	II	I	I
6	M	14	S.P	IV	IV	II	I
7	M	72	T.P	V	V	II	II
8	M	51	T.P	V	IV	III	III
9	M	37	S.P	IV	II	I	I
10	M	18	E.D	IV	I	I	I
11	M	76	S.P	V	IV	II	I
12	M	37	P.P	IV	I	I	I
13	M	41	T.P	IV	III	I	I
14	M	73	S.P	IV	IV	II	II
15	M	61	T.P	V	II	I	I
16	M	53	S.P	IV	II	I	I
17	M	76	E.D	IV	I	I	I
18	M	41	S.P	IV	III	II	II
19	M	55	S.P	IV	II	II	I
20	M	46	P.P	V	III	II	II
21	F	37	E.D	IV	II	I	I
22	F	68	T.P	IV	IV	II	I
23	F	71	S.P	V	II	I	I
24	F	48	P.P	IV	I	I	I
25	F	46	P.P	IV	II	I	I
26	F	47	S.P	IV	II	II	I
27	F	78	S.P	V	III	II	II
28	F	73	T.P	V	II	I	I
**Non-Kabat Group**							
1	M	45	P.P	IV	IV	III	I
2	M	36	S.P	V	V	III	II
3	M	65	P.P	IV	III	II	I
4	M	82	S.P	V	III	II	II
5	M	73	S.P	IV	IV	IV	I
6	M	16	E.D	IV	II	I	I
7	M	78	T.P	V	IV	II	II
8	M	54	T.P	V	III	III	III
9	M	39	S.P	IV	III	I	I
10	M	15	S.P	IV	II	I	I
11	M	78	T.P	V	IV	II	I
12	M	47	S.P	IV	II	II	I
13	M	29	S.P	IV	III	II	I
14	M	68	P.P	V	IV	III	I
15	M	57	T.P	V	III	II	II
16	M	66	S.P	IV	II	I	I
17	M	81	E.D	IV	III	II	I
18	M	33	P.P	IV	III	II	I
19	M	32	E.D	IV	IV	II	I
20	M	64	T.P	V	IV	III	III
21	F	19	E.D	IV	III	II	I
22	F	77	T.P	V	V	IV	II
23	F	65	S.P	V	IV	III	I
24	F	51	P.P	IV	III	II	I
25	F	32	S.P	IV	III	II	I
26	F	82	T.P	IV	IV	III	II
27	F	49	S.P	V	IV	IV	II
28	F	78	P.P	IV	II	I	I

**Table 3:** Linear regression model with all variables ^1^ (EoS refers to different extent of surgery: PP, partial parotidectomy; SP, superficial parotidectomy; TP, total parotidectomy).

Parameter	Estimate	St.Error	t-Value	Pr (>|t|)	Level of Significance
**Intercept**	1.405911	0.423367	3.321	0.001701	**
**Kabat**	−0.712216	0.202041	−3.525	0.000929	***
**Age**	0.008280	0.005551	1.492	0.142208	
**Gender**	−0.049406	0.225573	−0.219	0.827539	
**EoSPP ^1^**	0.449229	0.350219	1.283	0.205628	
**EoSSP ^1^**	0.567987	0.314315	1.807	0.076894	
**EoSTP ^1^**	0.593099	0.354328	1.674	0.100529	

** *p* ≤ 0.01; *** *p* ≤ 0.001; *p* > 0.05.

**Table 4:** Linear regression model with the final set of independent variables.

Parameter	Estimate	St.Error	t-Value	Pr (>|t|)	Level of Significance
**Intercept**	1.70734	0.31214	5.470	1.24 × 10^−6^	***
**Kabat**	−0.71161	0.20144	−3.533	0.000862	***
**Age**	0.01070	0.00514	2.082	0.042151	*

* *p* ≤ 0.05; *** *p* ≤ 0.001.

**Table 5:** Logistic regression model for binary data results.

Parameter	Estimate	St.Error	Z Value	Pr (>|z|)	Level of Significance
**Intercept**	−2.37459	1.19865	−1.981	0.0476	*
**Kabat**	−2.03370	0.85060	−2.391	0.0168	*
**Age**	0.03181	0.01929	1.649	0.0992	

* *p* ≤ 0.05; *p* > 0.05.

## Data Availability

Data are available upon reasonable request from the corresponding author (G.L.G).
